# Applying the Behavioural Change Wheel to Encourage Higher Welfare Food Choices

**DOI:** 10.3390/ani9080524

**Published:** 2019-08-02

**Authors:** Amelia Cornish, Jen Jamieson, David Raubenheimer, Paul McGreevy

**Affiliations:** 1 Faculty of Veterinary Science, University of Sydney, Sydney, NSW 2006, Australia; 2 Ministry for Primary Industries, PO Box 2526, Wellington 6140, New Zealand; 3 Charles Perkins Centre and School of Life and Environmental Sciences, University of Sydney, Sydney, NSW 2006, Australia

**Keywords:** attitude-behaviour gap, behaviour change wheel, COM-B model, consumer attitudes, consumer concerns, farm animal welfare, food purchase decisions, higher welfare products

## Abstract

**Simple Summary:**

Public concern for animal welfare in food production continues to grow. However, its growth does not correspond with the increase in demand for higher welfare products, giving rise to an outcome known as the attitude–behaviour gap. Addressing this attitude–behaviour gap and supporting consumers to make higher animal welfare choices in the supermarket can play important roles in improving the lives of farm animals. Despite increasing awareness in this area, solutions aimed at minimizing this gap often focus on knowledge transfer and have not yet had a significant impact. The aim of this article is to review current knowledge around the attitude-behaviour gap, and situate it within the context of the behaviour change wheel; exploring the capabilities, opportunities, and motivations driving, as well as the barriers preventing consumers from making higher welfare food choices. Using this framework, the review aims to broadly identify how consumers can be encouraged to change their behaviour and buy higher welfare products.

**Abstract:**

Over the last several decades, positive public attitudes towards animal welfare have continued to develop. Consumers’ attitudes towards farm animal welfare indicate increasing concern about animal welfare in food production. Yet, this growing interest in the lives of farm animals does not correspond with a wholesale increase in demand for higher welfare products, providing evidence of the citizen-consumer attitude-behaviour gap (herein referred to as the attitude-behaviour gap). Minimising the attitude–behaviour gap and supporting consumers to make higher animal welfare choices may help producers to enhance the lives of farm animals. However, despite increasing awareness in this area, solutions to resolve this gap often focus on knowledge transfer and do not appear to have had a significant impact. The aim of this article is to review current knowledge around the attitude-behaviour gap, and situate it within the context of the behaviour change wheel; exploring the capabilities, opportunities, and motivations driving, as well as the barriers inhibiting consumers from making higher welfare food choices. Using this framework, the review aims to identify interventions that may boost consumer demand for higher welfare products sold at a premium price and provide suggestions for future research. Further work to increase understanding in this area is then also suggested.

## 1. Introduction

Arguably, the world’s current greatest challenge is how to sustain the almost 10 billion people who are expected to populate it by 2050 [1,2,3]. With global population growth, household incomes around the world are rising and people are eating more animal-based foods [1,2,3]. In parallel, the production of food from animals has moved away from small scale and extensive operations towards intensive systems characterised by confinement. Such intensification has resulted in an abundance of inexpensive animal-based foods, but it has also come at a great cost to production animal welfare [2,4].

Growing public concern for the welfare of farm animals is well documented [5,6,7,8], with evidence of consumers’ self-reported willingness to pay premium prices for food produced under high animal welfare standards [8,9]. However, these increasingly positive attitudes regarding the lives of farm animals [10] do not manifest reliably as higher welfare purchase decisions or behaviours [11,12,13]. A number of reasons have been offered to explain the disparity between consumers’ attitudes (or stated concern for production animal welfare) and their actions at the checkout, a common mismatch known as the attitude-behaviour gap [14,15], many of which will be explored in detail in this review.

Traditional interventions aimed at addressing the attitude-behaviour gap associated with ethical concerns, such as farm animal welfare, have focused on knowledge-transfer [16]; assuming that once consumers become aware of issues such as animal welfare they will change their behaviours and, in this case, begin to engage in higher welfare purchase decisions. However, much social science research has indicated that knowledge-transfer largely fails to change behaviours [16]. To identify interventions that are expected to be effective in changing behaviour, it is important to use a deliberate system to both consider and select from the full range of options available, and so avoid overlooking important alternatives. In 2011, Michie and colleagues [17] developed the human behaviour change wheel (BCW) as an example of such a system. The wheel provides a comprehensive tool allowing a range of users to design and select interventions and policies according to an analysis of the nature of the behaviour and the mechanisms that need to be changed to bring about behaviour change [17].

The objective of the current review is to summarise current interdisciplinary knowledge around the attitude-behaviour gap for farm animal welfare, situating it within the framework of the BCW. In particular, it will address the capabilities, opportunities, and motivations driving, as well as inhibiting, consumers when making higher welfare food choices. Given that animal welfare improvements in food production are increasingly market-driven, the BCW is used to identify interventions with the potential to support and drive consumer purchase of higher welfare products. In light of growing societal concern for animal welfare [10], now is an opportune time and context to explore such behaviour change interventions.

## 2. A Brief Introduction to the Attitude-Behaviour Gap for Farm Animal Welfare

We begin our discussion regarding public concern for farm animal welfare by addressing the question: What is animal welfare? Although there remains no single globally accepted definition, three main scientific concepts are widely used to evaluate the status of an animal’s welfare. These are biological function, affective state, and natural living. The first of these emphasises the biological functioning of animals, such as reproduction; the second addresses the psychological aspects of welfare and the mental experiences of animals; while the third highlights the need for animals to live naturally and to have the ability to express innate behaviours [18]. Only once we have stipulated which of these concepts is most relevant in a given case, can the welfare of an animal be evaluated.

Increasing public concern for farm animal welfare is well documented [19,20], but do consumers necessarily express this concern in their market behaviour? The evidence is to the contrary. Despite their best intentions to be ethical, consumers rarely purchase ethical products at the checkout, providing evidence of an attitude-behaviour gap [14,15,21,22,23,24,25,26]. By way of an example from another area of concern, 30% of UK consumers reported that they were very concerned about environmental issues; yet only 5% translated this into ethical food choices at the checkout [13]. Such disparity has also been referred to as a consumer-citizen gap, with scholars arguing that there is a gap between the responsible intentions of citizens and the hedonic desires of consumers [25,27].

Empirical evidence supports the notion of an attitude-behaviour gap for animal welfare. Bennett [28] found that 86% of European respondents reported being concerned about farm animal welfare, but only 61% stated that they purchased higher welfare products. A study across France, England, Sweden and Denmark found although respondents considered modern animal production as inhumane, they freely admitted, in focus group discussions, that their concerns and exposure to negative images of production methods did not influence their purchase decisions [29]. Also, Mayfield et al. [30] found that 77%, 50% and 56% of respondents from Italy, Great Britain and Sweden, respectively, reported that farm animal welfare was important to them, but only 41%, 23% and 25%, respectively, reported always thinking about animal welfare when buying meat. More recently, Cornish et al. [31] found that despite 92% of Australian respondents describing animal welfare as “important” to them when purchasing animal-based products, only 76%, 53%, and 31% reported buying welfare-friendly eggs, poultry and pork, respectively. A key limitation of the published research on the attitude-behaviour gap is the general reliance on self-reported data, such as that discussed above, which cannot exclude the possibility of respondents providing false information. Similarly, self-reported data can represent respondents’ conflated motivation when they are influenced by social-desirability bias or a tendency to answer questions in a manner that will be viewed more favourably by others.

## 3. The Behaviour Change Wheel for Understanding Behaviour

**Figure 1:**
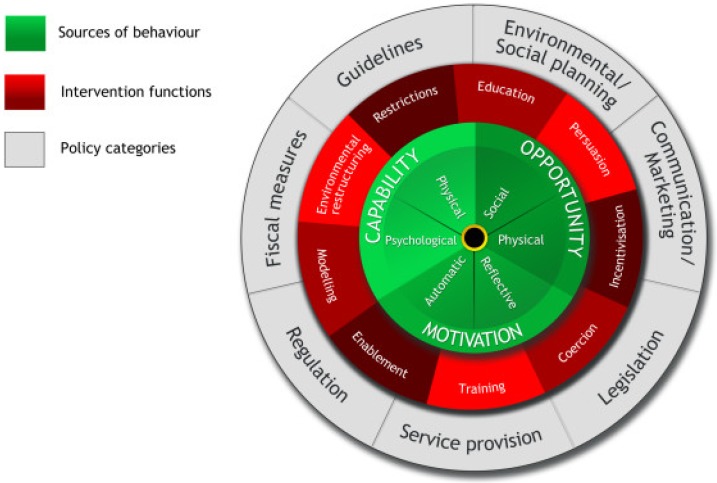
The behaviour change wheel (BCW) showing at the core the capabilities (C), opportunities (O), and motivations (M) that are considered sources of behaviour. Then, the outer two layers of the wheel that outline the ways that sources of behaviour can be influenced through nine possible intervention functions (listed above) and, finally, seven policy categories (also listed above) (reproduced with permission, after Michie et al. [17]).

The BCW developed by Michie and colleagues provides a structured approach to designing behaviour change interventions and strategies [17]. At the centre of the wheel, capability (C), opportunity (O), and motivation (M) are proposed as interacting determinants of behaviour (B) (hence COM-B model) (see Figure 1 below) [17]:

1. Capability reflects an individual’s psychological and physical ability to engage in the behaviour of interest. Physical capability refers to the extent to which an individual can engage in the behaviour, for example, if constrained by financial resources. Psychological capability refers to the individual’s capacity to engage in the required cognitive processing.
2. Opportunity comprises the factors external to an individual that are required for the behaviour to occur. This can either mean physical opportunity, or situational factors and social opportunity, i.e., the cultural or community values and norms that encourage or deter certain behaviours.
3. Motivation encompasses factors internal to an individual, such as brain processes, that energize and direct behaviour. Such intrinsic factors include conscious decision-making, habitual processes, and emotions. The COM-B model distinguishes two types of motivations that are reflective processes, which include plans (intentions) and evaluations (beliefs about what is desirable and undesirable), and automatic processes that include emotions, desires and impulses that arise from associative learning.

Depending on the behavioural determinant to be focused on, there are nine possible intervention functions to choose from: Education; persuasion; incentivisation; coercion; training; restriction; environmental restructuring; modeling; and enablement. Seven policy categories are then presented to support the delivery of the particular intervention functions: Communication/marketing; guidelines; legislation; regulation; fiscal, environmental/social planning; and service provision.

## 4. Purchasing Behaviour for Higher Welfare Products; Capability, Opportunity and Motivation

Literature is reviewed below where it provides insight into consumers’ purchasing decisions around higher welfare products in the supermarket.

### 4.1. Capability: Physical

#### Inability to Afford the Products

Despite some inconclusive findings [32], most research into ethical consumerism suggests that price trumps ethical concerns [23,33]. In the context of animal welfare, this holds true [34,35]. Higher welfare products are sold at a price premium compared to conventional products and, for many consumers, financial constraints may inhibit their ability to purchase the higher welfare product. The price premium (or higher cost) of higher welfare production is due to the allocation of more space and infrastructure per animal and greater care and stockperson time; production costs that are passed on to the consumer. Recently, Vigors [36], in a review exploring the effect of nudging behaviours to reduce the attitude-behaviour gap for farm animal welfare, noted that the relative price of products, rather than their absolute price influences consumer purchases. So, consumers are affected by how the price of one product compares to the price of another, e.g., the higher price of free-range eggs compared to conventional caged eggs.

### 4.2. Capability: Psychological

#### 4.2.1. Lack of Awareness

As mentioned earlier, there is no single globally accepted definition for animal welfare and scientists continue to debate what systems of production are preferable, for example free-range versus enriched caged egg production systems. Given that experts are not in agreement, it should not be surprising that the general public as consumers are confused and generally have a poor understanding of what constitutes a higher welfare product. Moreover, misleading or overwhelming labels about animal welfare often only make matters worse and inhibit higher welfare purchasing. Consumers are seemingly overwhelmed by the proliferation of mandatory and voluntary labels or claims on food packages [11,27,37,38,39]. For example, a 2007 review of the welfare-friendly food labels across six European countries questioned the transparency of information provided [40]. In Australia, a recent report commissioned by the Federal Government found that 42% of respondents stated that there is too much conflicting information about animal welfare and 40% felt they did not have enough information to understand what happens in the agricultural industry [41].

#### 4.2.2. Difficulty Processing Available Information

As humans, we are bounded by the limits of our own rationality. This means that our ability to seek out further information in our decision-making is limited by the time afforded to us, and also by our analytic power or ability to process information and choose between options [36,42,43]. Consumers currently face ever-increasing numbers of choices and product differences when selecting animal-based foodstuffs with different labels, such as free-range, cage-free, hormone-free, to name a few [27,44,45,46,47]. Despite the best intentions of many producers to educate and inform consumers, consumers are often constrained in their ability to use such additional information to help them make higher welfare purchase decisions [36,42,43].

#### 4.2.3. The Curse of Willful Ignorance

Many consumers do not always want to be informed, preferring to remain willfully ignorant [48]. Wilful ignorance is a form of avoidance behaviour likely to be reinforced by two factors. Firstly, the higher than conventional welfare purchase decision brings with it an opportunity cost in that the complexity of animal welfare information can mean that processing the core information requires time and effort; time and effort that therefore cannot be expended elsewhere. As such, consumers may decide that processing such information is not worth the effort [49]. Secondly, there are low error costs associated with poor welfare choices [50], so any consequences or repercussions of not purchasing higher welfare options are low or non-existent. A European study into farm animal welfare found that when asked how often they think about farm animal welfare when buying meat, only 41%, 23% and 25% of consumers in Italy, Great Britain and Sweden, respectively, reported always thinking about it, with many stating they did not like to think that meat came from an animal [30].

#### 4.2.4. Abrogation of Responsibility to Others

Consumers may consider boosting animal welfare in food production to be a “public good” which should be provided to the community by governments. Therefore, they may not actively seek and demand high welfare products but instead default to assume maintaining, monitoring and elevating farm animal welfare standards to be the responsibility of regulators. Currently, improvements to farm animal welfare are market-driven, thus it could be argued animal welfare is primarily a responsibility of consumers to act in accordance with their ethical concerns.

### 4.3. Opportunity: Physical

#### Poor Availability

Perceived (or real) low availability of higher welfare products has been suggested as a cause of the attitude-behaviour gap [27,30,37,39]. As an example, Vermeir and Verbeke [12] found that, despite consumers stating an intention to buy sustainable dairy products, they often did not because such products were not widely available. More recently, Cornish and colleagues [31] surveyed Australian purchasing of a small range of welfare-friendly food products and found that while 58% of respondents reported there being sufficient welfare-friendly eggs available to them when shopping, 50% and 63% reported that there was not sufficient welfare-friendly poultry and pork, respectively, available to them.

### 4.4. Opportunity: Social

#### Social Pressures

Food purchasing and consumption can be considered a social act in that they are intertwined in the human psyche and, often without even knowing it, we change how and what we eat based on the people around us. Social norms represent the implicit or explicit rules and expectations of society. They amount to one’s beliefs about what other people think one should or should not do [36,51]. Social norms have been found to strongly shape meat consumption [51]. Indeed, Schenk, Rössel and Scholz [51] found that ethical motivation was more strongly influenced by social norms than restricted by convenience and taste. Napolitano and colleagues [52] also suggest that social norms are a probable cause of the attitude–behaviour gap for farm animal welfare. Therefore, it may be that although pro-welfare social norms exist, they do not positively influence consumer behaviour [52]. Moreover, the power of social norms can have a positive influence on situations requiring heavier cognitive loads, with individuals often using the behaviour of others as a guide for what they should do [36]. Additionally, some scholars have argued that food choices, particular for animal-based products, are governed by dominant sociocultural norms. For example, Jenkins and Twine [53] contend that vegans, and some vegetarians, are going against the dominant norm. However, in the wake of the recently released government-funded report into Australian attitudes to animal farming, it is overwhelmingly clear that public understanding of and concern for farm animal welfare has changed over time, with 95% of respondents concerned about farm animal welfare and 91% calling for regulatory reform [41]. Thus, it could be contended that the day when such dominant norms change has arrived and the animal welfare-friendly or even plant-based alternative are becoming dominant.

### 4.5. Motivation: Reflective

#### 4.5.1. Intentions to Purchase Higher Welfare

Reflective processing involves the creation of self-conscious intentions, such as intending to purchase higher welfare products. Research into consumers’ attitudes towards animal welfare in food production has increased in the last decade or so, with notable findings from Australia, [5,6,8], the European Union (EU), [7,19], and the United States of America (USA), for example [54]. As an illustration, an Australian survey found that 71% of respondents agreed that farm animal welfare is an important consideration, and 62% disagreed that demand for food was more important than the humane treatment of animals [5].

Moreover, reflective processing involves evaluations about what is good and bad. People concerned about animal welfare issues have been shown to hold positive attitudes towards higher welfare products [55]. However, behavioural economic research suggests consumer preferences are rarely stable; rather they vary between and within decisions [36]. This is true for animal welfare, with consumers being shown to exhibit heterogeneous, unstable preferences for animal welfare attributes of food products [49,56,57,58,59]. As an example, consumers may express concern for animal welfare but this may not be the most important factor influencing their purchase decisions (with more salient issues, such as price, superseding animal welfare concerns), or they purchase higher welfare eggs but not pork. Moreover, animal welfare, as a food attribute, cannot be directly observed or evaluated by a consumer, even after purchase, so its potential to differentiate between products is only small [46,49,59,60,61]. Also, it is worth noting that ethical and unethical information has an asymmetrical influence on attitudes, with vices (or bad and unethical attributes) detracting from the engagement in ethical actions more than virtues (or good and ethical attributes) increase them. Therefore, it could be assumed that consumers are more willing to punish or boycott unethical brands or products, rather than reward or purchase ethical ones [38]. For example, a Norwegian study into consumers’ perceptions of animal welfare labelling when buying eggs found that negative labelling compromised egg sales, whereas positive labelling regarding good welfare did not [62].

#### 4.5.2. Additional Drivers Other Than Concern for Animal Welfare

Consumers have been found to have motivations to buy higher welfare products other than, or in addition to, animal welfare concerns. It has been proposed that they consider animal welfare not just within an ethical context, but also as part of more traditional notions, and that they associate the well-being of farmed animals with the quality, food safety, provenance, taste and healthfulness of foods [39,44,60,63,64,65,66,67]. A recent Australian study by Bray and Ankeny [67] found that consumers perceived free-range and cage-free eggs as being of higher quality, more nutritious, safer, and having better sensory characteristics, than caged eggs (regardless of whether these claims are true or not).

The main factors found to drive consumer purchase decisions regarding higher welfare products include:

- Food safety: A US study found more than three-quarters of respondents agreed, “animals raised under higher standards of care will produce safer and better tasting meat [54]”.
- Quality: Consumers do not accept ethical corporate behaviour as a substitute for product quality [68] and they associate higher animal welfare production with better quality products [30,64].
- Provenance: Consumers prefer local products, perceiving products from their own country to be higher quality perhaps due to the assumption of higher animal welfare standards, irrespective of whether this is actually the case [29,34,60]. Davidson et al. [69] found that 77% of Scottish consumers considered country-of-origin the most important food attribute when purchasing meat.
- Health: Consumers associate animal welfare with the healthfulness of products [30,66]. Research found UK consumers perceived welfare-friendly products as safer and healthier [39]. For example, 78% of UK respondents thought welfare-friendly meat was healthier and 71% thought it had more nutritional value [70].
- Palatability: Consumers expect welfare-friendly products to taste better [60,71]. A recent study by Malone and Lusk [72] found that taste is the most important meat product perception; superseding healthfulness and safety.

### 4.6. Motivation: Automatic

#### 4.6.1. Habitual Processing

Food acquisition is generally governed by habitual purchasing behaviour [12,23]. Consumers do not make trade-offs between food attributes at every purchase, but rather recall the result of an earlier trade-off and repeat the decision [73]. The automatic, unconscious nature of habitual behaviours requires very low engagement or awareness. In contrast, ethical purchasing requires time and effort [12,73,74] and consumers who are fully informed to make effective purchasing decisions that align with their values (Sproles et al. 1978, as cited in [23]).

#### 4.6.2. Self-Identity

Food purchase and consumption are embedded within our own personal psyche and hold meaning to us [51] and often play a large role in our own self-identity. For example, Schenk and colleagues [51] found that having a vegetarian self-identify strongly determined meat-avoidance behaviour. Thus, consumers seeing themselves or wanting to be seen by others as someone who makes higher welfare choices will have their decisions driven by these considerations.

## 5. Recommended Interventions to Minimise the Attitude-Behaviour Gap

Current understanding of the attitude-behaviour gap, situated within the BCW framework, suggests that a range of interventions should be explored to better align consumers’ behaviour with their pro-welfare intentions. Table 1 shows the links between the components of the BCW and the nine possible intervention functions [17]. Within the framework each intervention is associated with a number of commonly used behaviour change techniques. This paper does not aim to provide an exhaustive list of all approaches that could be taken, but rather to highlight a few examples.

### 5.1. Education

Education involves increasing knowledge and understanding [17]. To educate consumers, stakeholders must not only aim to raise awareness around animal welfare concerns, but that they also address the motivations other than or in addition to animal welfare concerns that have been found to drive consumer purchase decisions, e.g., product quality, food safety, taste and healthfulness [39,44,60,63,64,65,66,67]. Educational campaigns using in-store poster or placards that read along the lines of “free-range eggs not only taste better, but they are better for the hens too by allowing them more freedom to move” may be of benefit.

### 5.2. Persuasion

There may be merit in the use of campaigns in which pleas are made to “buy animal-friendly” or “be kinder to hens and buy free-range eggs”. Moreover, given food consumption and purchase is governed by social norms [51], thus informing consumers about what their peers are doing could also be effective in driving higher welfare choices. For example, using descriptive norms on poster or placards in-store, such as “two out of every three shoppers at (a named supermarket) buy cage-free eggs”.

### 5.3. Incentivisation

The creation of expected rewards for making animal-friendly choices could incentivise relatively high welfare purchase behaviours, with supermarkets or salient groups (such as sports clubs and local community centres) using prize draws, for example, everyone who buys cage-free eggs at (a named supermarket) enters the draw.

### 5.4. Coercion

Coercion involves creating the expectation of punishment or cost [17]. An effective example of creating expectation of punishment or cost for adopting an undesired behaviour is a tax rise on alcohol in an attempt to reduce excessive alcohol consumption. However, in the case of farm animal welfare, welfare increments come at a price premium. Governments could play a role in incentivizing farmers to optimise animal welfare either financially via subsidies or by other means, such as affording them priority access to resources (such as animal feed) or commercial opportunities. Additionally, governments could enforce sanctions for poor welfare. Nonetheless, the current market-driven nature of farm animal welfare makes this intervention somewhat counterintuitive because, in a sense, consumers may consider themselves to be paying a cost penalty for choosing relatively high welfare food. Addressing the price premium of higher welfare products is difficult (at least in the short term). However, consumers could be coerced through the threat of social punishment or social sanctions by peers or members of the community for not doing the animal-friendly thing.

### 5.5. Training

Training refers to imparting skills that can be put into action, for example driver training [17]. Training could be achieved with celebrities, educators or other members of the community demonstrating to consumers where to go to buy higher welfare production and which specific labels to look for.

### 5.6. Restrictions

Stakeholders interested in the sale and promotion of relatively high welfare products could help boost consumers’ understanding of what constitutes a higher than conventional welfare product and clarify labeling with repudiation of false claims and stronger governance over misleading marketing. For example, due to the opaque and misleading term “outdoor-bred” on pork products used in reference to piglets born outdoors, Australia’s leading consumer watchdog group pushed for the inclusion of the words “raised indoors on straw” to make it clearer to consumers that when weaned (at about 21 days) piglets in these systems are transferred to sheds indoors.

### 5.7. Environmental Restructuring

Environmental restructuring refers to efforts to change the physical or social context in which behaviours take place [17]. Time constraints and heuristic processing govern food-purchasing in-store behaviours. Furthermore, consumers can be affected by external factors such as supermarket lay-out and presentation of information [75], thus restructuring the physical purchasing environment to encourage pro-animal welfare purchases could be achieved through redesigning in-store product placement and store layout, for example, placing the free-range eggs on the shelf at the consumers’ eye-level and the caged eggs on the bottom shelf. Additionally, grocery stores could group higher welfare products together, similar to a “health food aisle”; there could be an “animal-friendly” aisle. Such efforts would also help address actual or perceived issues around the availability of relatively high welfare products.

Addressing the social context of purchasing animal-based products would be notably more difficult than addressing the physical context of such purchasing. However, social influence has been shown to affect behaviour with regards to issues such as binge drinking (e.g., [76]). Within the current context, creating a social environment wherein displaying support for farm animal welfare and buying the higher welfare food options are seen as the preferable behaviours could target the social context.

### 5.8. Modeling

Celebrities often provide suitable role models that people aspire to be like or imitate. In regards to animal welfare, the efforts of celebrities such as chef Jamie Oliver can educate and encourage consumers to purchase higher welfare products. Mass media such as television campaigns have been proven to influence consumer knowledge [30,34]. Jamieson et al. [77], when surveying adolescences about their attitudes to farm animal welfare found that television was the most common farm animal welfare information sources cited.

### 5.9. Enablement

Enablement refers to increasing means or reducing barriers to increase capability or opportunity [17]. Consumers could be enabled to buy higher welfare products by the development of a single national trusted certification and labelling process that helps consumers differentiate animal products based on the animal welfare conditions under which they were produced. The 2019 Australian Futureye report found that most respondents assumed that products labeled as cage-free, free-range, and organic reflected better animal welfare standards, but did not always trust these labels. One of the solutions to this that was raised in focus group discussions was a trusted certification and labelling process to help consumers differentiate products [41]. Such labelling systems could be endorsed by various stakeholders such as governments, non-government organisations (NGOs) or retailers. The success of any labelling initiative would depend on the auditing process, the frequency and transparency of which would be key for consumer trust, since verification of the labels’ claims is essential. This could also help address perceived (or real) issues around availability, as well as limiting consumers’ ability to engage in wilful ignorance of animal welfare concerns when shopping in the supermarket, with pivotal information becoming less easy to disregard.

## 6. Discussion

The evidence outlined in our review suggests that consumers are increasingly concerned about animal welfare, but such sentiments are not readily converted to real actions at the supermarket checkout, with indication of an attitude-behaviour gap. Consumer food purchasing decisions are complex and involving a range of factors beyond animal welfare concerns alone, with animal welfare often being considered in parallel to many other individual food attributes, such as quality and taste.

Given animal welfare improvements in food production are market-driven, great improvements in farm animal welfare can be achieved by increasing consumer demand for welfare-friendly products. Interestingly, the 2019 Futureye report found that 65% of Australian respondents were willing to pay more to ensure better animal welfare standards [41]. Such pro-animal welfare intentions suggest a current market failure; with consumers not being supported or enabled in their desire to better align their attitudes and actions. It is clear from the current review that there is no silver bullet, and efforts to encourage consumers to purchase higher welfare products will require a multi-pronged and integrated approach involving various stakeholders from the Government and major supermarkets to everyday individuals.

Changing behaviour is difficult, and sustaining change is even harder. Behaviour change interventions are likely to fail if they try to change too many behaviours at once. It should be noted that this review focussed broadly on literature surrounding consumers buying high welfare produce, rather than addressing a specific scenario such as purchases of relatively high welfare poultry meat in Australian Supermarkets. This was intentional as a means to open up a general dialogue around using the BCW to assist in closing the attitude-behaviour gap. However, whilst this exploration has added to the growing literature in this area, it has not provided a definitive guide of what exactly needs to change to close the gap. To do so, the broad issue of the attitude-behaviour gap needs to be broken down and defined as a range of more specific behaviours that can be addressed. To achieve this, we suggest future research focuses on how different categories of consumers respond to various interventions. Further narrowing of the focus to define the problem in terms of a specific behaviour, i.e., the purchasing of an identified product or species, e.g., dairy products, followed by analysis of the targeted population (using the COM-B approach), will allow identification of distinct categories of consumers. With this insight, tailoring of messaging and matching of specific interventions to each audience’s needs can then progress. As understanding increases, a database could be created to analyse what works best for each category, rather than commenting on consumers broadly, as a uniform population as we have done.

## 7. Conclusions

In conclusion, examination of the attitude-behaviour gap for farm animal welfare confirms that consumers’ sentiments for animal welfare are not reflected in their purchase behaviour. Our exploration of the gap using the BCW framework reveals the various drivers and barriers that consumers encounter when considering relatively high welfare food choices. We argue that while many consumers do intend to consume ethically, various purchase barriers hamper them and competing demands get in the way before they get to the checkout. For example, despite their concerns for animals, they care more about price.

Accordingly, we have provided many examples of the interventions that could be used to boost consumer demand for relatively high welfare products. By enabling consumers to engage in more pro-animal welfare behaviours farmers can improve the welfare of millions of animals produced for food each year. Undoubtedly, there is not one single answer when it comes to encouraging consumers to purchase relatively high welfare products. Instead, it will require a multi-faceted approach that addresses the many barriers outlined above.

## Figures and Tables

**Table 1:** The relationship between the components of the ‘COM-B’ model of behaviour (capabilities, opportunities, and motivations) and the nine intervention functions (adapted from Michie et al. [17]). A tick indicates where a particular intervention function could be used to address each component of the BCW.

Model of Behaviour Source		Education	Persuasion	Incentivisation	Coercion	Training	Restriction	Environmental Restructuring	Modeling	Enablement
Capability	Physical					√				√
	Psychological	√				√				√
Motivation	Reflective	√	√	√	√					
	Automatic		√	√	√			√	√	√
Opportunity	Physical						√	√		√
	Social						√	√		√
